# Therapeutic potential of chemokine signal inhibition for metastatic breast cancer

**DOI:** 10.1016/j.phrs.2015.08.004

**Published:** 2015-10

**Authors:** Takanori Kitamura, Jeffrey W. Pollard

**Affiliations:** a MRC Centre for Reproductive Health, The Queen's Medical Research Institute, The University of Edinburgh, 47 Little France Crescent, Edinburgh EH16 4TJ, UK; b Department of Developmental and Molecular Biology, Albert Einstein College of Medicine, 1300 Morris Park Avenue, Bronx, NY 10543, USA

**Keywords:** MAM, metastasis-associated macrophages, CSF-1, colony stimulating factor-1, VEGF, vascular endothelial growth factor, CCL, CC-chemokine ligand, CCR, CC-chemokine receptor, IM, inflammatory monocyte, CXCL, CXC-chemokine ligand, CXCR, CXC-chemokine receptor, Breast cancer, Metastasis, Macrophage, Chemokine

## Abstract

Metastatic breast cancer is incurable by current therapies including chemotherapy and immunotherapy. Accumulating evidence indicates that tumor-infiltrating macrophages promote establishment of the lethal metastatic foci and contribute to therapeutic resistance. Recent studies suggest that the accumulation of these macrophages is regulated by a chemokine network established in the tumor microenvironment. In this perspective paper, we elaborate on the chemokine signals that can attract monocytes/macrophages to the site of metastasis, and discuss whether inhibition of these chemokine signals can represent a new therapeutic strategy for metastatic breast cancer.

## Graphical abstract

Tumor-infiltrang immune cells such as metastasis-associated macrophages (MAM), regulatory T (Tregreg) cells, and myeloid-derived suppressor cells (MDSC) are reported to promote establishment of the lethal metasta-c foci and restrict efficacy of cytotoxic drugs or tumoricidal immune responses by natural killer (NK) and CD8+ T cells. Recent studies suggest that these pro-tumor immune cells are accumulated a chemokine network established in the tumor microenvironment. Therefore, blockade of these chemokine signals could improve therapeu-c efficacy of chemotherapy and immunotherapy.

**Figure:**
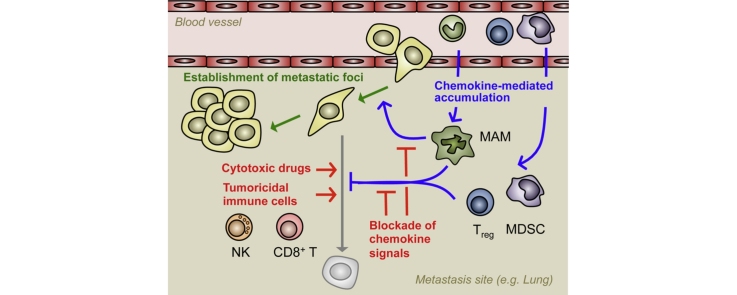

## 1 Introduction

Breast cancer is a leading cause of cancer death in women largely due to metastasis that develop in the bone and lung. The 5-year survival of patients with metastatic disease drops to 21% whereas that of patients with early-stage breast cancer is 89–100% [1]. It has been reported that 6–10% of breast cancer patients are found to have metastasis at initial diagnosis and ∼30% of tumor-resected patients develop distant metastases [2], indicating the failure of current therapies and requirement of novel strategies to prevent tumor growth at the metastatic sites. Among potential new therapeutic targets are stromal cells, especially macrophages, within the tumor microenvironment as they promote establishment of the lethal metastatic tumors [3–6] and prevent the efficacy of current therapies [7–9].

In breast cancer mouse models, lung metastatic foci show marked accumulation of a distinct macrophage population (F4/80^+^CD11b ^+^Ly6C^–^) that is barely found in the normal lung [10] . In an experimental model of pulmonary metastasis, these metastasis-associated macrophages (MAMs) are recruited to the lung and directly contact with disseminating mammary tumor cells within 24–48 h post-tumor injection, which enhances extravasation of the circulating tumor cells and suppresses tumor cell apoptosis [10,11]. Since genetic depletion of these CD11b^+^ macrophages reduces the metastatic tumor burden in the lung [10], blockade of the MAM accumulation is an attractive therapeutic strategy for metastatic breast cancer.

It has been reported that the recruitment of macrophages to the primary site is promoted by various cytokines and chemokines such as colony stimulating factor-1 (CSF-1), vascular endothelial growth factor (VEGF) and CC-chemokine ligand 2 (CCL2), although the mechanisms underlying macrophage accumulation in the metastasis sites are still largely unknown. We have recently reported that the accumulation of MAMs at the metastatic lung is regulated by chemokine ligands CCL2 and CCL3 and their respective receptors CCR2 and CCR1 [12,13]. In this perspective paper, we describe the roles of these chemokine signals in the MAM accumulation, and discuss therapeutic potential of their blockade for metastatic breast cancer.

## 2 Accumulation of metastasis-associated macrophages via chemokine signals

**Fig. 1:**
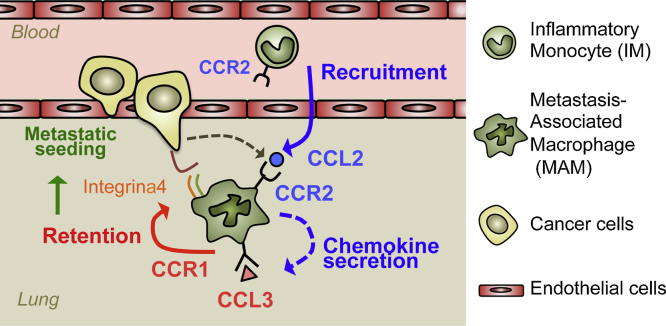
Model for a chemokine cascade that promotes metastatic seeding of cancer cells. Cancer or host cell derived CCL2 promotes recruitment of inflammatory monocytes (IMs) from circulation to the metastasis sites. These recruited IMs differentiate into metastasis-associated macrophages (MAMs) that express higher levels of CCR1. In the MAMs, CCL2-CCR2 signaling increases expression of CCL3. CCL3-CCR1 autocrine signaling enhances and stabilizes cancer cell-MAM interaction in part through integrin α4 binding to VCAM1 expressed on the tumor cell [11]. This results in the retention of MAMs that further promotes metastatic seeding of cancer cells through at least in part conferring survival signals on the metastatic cell. Blue and red arrows show CCL2 and CCL3 mediated events respectively. Dotted lines indicate secretion of chemokines.

It has been reported that high levels of CCL2 in breast cancer specimens correlate with high number of macrophages in the primary tumors [14], suggesting pivotal roles of CCL2 in macrophage recruitment to the tumor microenvironment. We recently demonstrated that anti-CCL2 antibody treatment decreases the number of MAMs at the metastatic sites and reduces metastatic tumor burden in an experimental model of breast cancer lung metastasis [12]. In this model, anti-CCL2 antibody treatment also inhibited the migration of adoptively transferred inflammatory monocytes (IMs; CD11b^+^Ly6C^high^) to the tumor challenged lung. These results indicate that the CCL2-CCR2 signal recruits circulating IMs to the site of metastasis where they differentiate into MAMs and promote establishment of metastatic foci. We have further found that the MAMs isolated from the mouse lung with metastatic foci express much higher level of CCL3 compared with circulating IMs [13]. Interestingly, the CCL3 expression in MAMs is significantly suppressed by anti-CCL2 antibody treatment, suggesting that MAMs secrete high level of CCL3 once they differentiate from IMs and this is partly through activation of the CCL2-CCR2 signaling pathway. Genetic loss of host CCL3 or its receptor CCR1 reduces the MAM accumulation in the tumor-challenged lung 24 h after tumor injection and decreases number of metastatic foci. Loss of CCR1 also prevents MAM-cancer cell interactions and following retention of MAMs in the tumor-challenged lung. These findings collectively indicate that activation of the CCL3-CCR1 axis in MAMs via CCL2 signaling promotes retention of MAMs and subsequent metastatic seeding of breast cancer cells (Fig. 1). These results suggest that distinct chemokine receptors regulate specific process of the monocyte/macrophage accumulation, i.e., recruitment by CCL2-CCR2 and retention by CCL3-CCR1 axis. Consistent with this suggestion, in vitro studies show that migration, adhesion, and differentiation of human monocytes are promoted by CCL2, CXCL18, and CXCL12 respectively [15–17].

In our breast cancer model, another CCL3 receptor CCR5 is not necessary for the early MAM accumulation observed within 24 h after tumor injection. However, it is reported that CCR5 is required for macrophage accumulation in the lung foci after 7 days of renal cancer cell injection [18], suggesting that macrophages use different chemokine receptors to accumulate in the distinct microenvironments at different phases of metastasis. Recent studies utilizing the PyMT mice suggest that a chemokine receptor predominantly used for macrophage accumulation might be skewed by the induction of a specific ligand in the tumor microenvironment. Namely, increased CCL2 level in the tumor by doxorubicin treatment promotes CCR2 dependent monocyte recruitment [19], although the PyMT mammary tumors normally recruit monocytes/macrophages via CCR6 but not CCR2 [20,21] . Induction of certain ligands in cancer cells as they progress may also determine the chemokine receptor required for the macrophage accumulation since the primary tumors developed by mouse breast cancer cells that overexpress CXCL12 or CX3CL1 recruit macrophages through CXCR4 or CX3CR1 respectively [22,23]. In the primary tumors developed by 4T1 mouse breast cancer cells, inhibition of either CCR2 or CXCR2 can reduce the number of macrophages [24,25], which also suggests the involvement of multiple chemokine signals in the macrophage accumulation in the tumor microenvironment. However, most of these findings come from in vitro systems or primary tumor models. Further studies are required to evaluate the involvements of these chemokine signals other than CCL2 and CCL3 in the MAM accumulation at the metastasis sites, as their actions might represent therapeutic targets for metastatic diseases.

## 3 Inhibition of chemokine signals to prevent metastatic outgrowth of beast cancer cells

The ultimate objective of macrophage-targeting therapy is withdrawal of tumor-supporting and immunosuppressive microenvironment from the secondary sites by disrupting accumulation and/or function of MAMs. Accordingly, the above-mentioned chemokine signaling molecules are potential targets for the treatment of metastatic breast cancer.

Results from our breast cancer metastasis model suggest that the inhibition of CCL3 secretion from MAMs is one of the possible strategies as they are a major source of CCL3 among other leukocytes such as neutrophils, T, B, and NK cells in the metastatic lung [13]. CCL2 is another possible target fitting this strategy since CCL2 neutralizing antibody can suppress *Ccl3* expression in MAMs as well as their recruitment following mammary tumor metastasis [12,13]. However, humanized monoclonal CCL2 neutralizing antibody (CNTO888) is ineffective in suppressing serum CCL2 level or tumor progression due to feedback mechanism that increases CCL2 production [26]. Furthermore, discontinuing anti-CCL2 treatment is reported to cause rebound influx of monocytes into the metastatic sites that enhances metastatic outgrowth [24]. These reports suggest difficulty in suppressing MAM accumulation by CCL2 deprivation, and indicates requirement for another target. In our model, the anti-CCL2 antibody treatment reduces CCL3 expression in MAMs but inhibition rate is only 40% compared with IgG treatment [13], suggesting that factors other than CCL2 from cancer cells and/or tumor microenvironment also involve in CCL3 secretion from MAMs. It has been reported that CCL3 expression in bone marrow-derived macrophages is increased by granulocyte- macrophage colony-stimulating factor (GM-CSF), IL-3 and IL-33 [27,28]. Interestingly, chemokine ligands such as CCL5 and CCL18 can also promote secretion of various chemokines including CCL3 from cultured human monocytes [29,30]. These cytokines and chemokines could be alternative targets to suppress CCL3 secretion, although further studies are required to understand their expression in metastatic sites and their contribution to MAM accumulation.

Another possible strategy to suppress MAM accumulation is blockade of CCR1 and CCR2. Several companies have developed small molecule inhibitors against CCR1 or CCR2 for rheumatoid arthritis or multiple sclerosis, and most of them are well tolerated and show no adverse effects [31]. These antagonists were developed for autoimmune diseases, and thus clinical trials for cancer are very limited. Nevertheless, an anti-CCR2 antibody (MLN1202) tested in a phase II clinical trial for metastatic cancer showed therapeutic effects in 14 out of 43 patients with bone metastases (ClinicalTrials.gov ID: NCT01015560). Most recently, Chemocentryx initiated a phase Ib trial of their next-generation CCR2 antagonist (CCX872) for non-resectable pancreatic cancer (ClinicalTrials.gov ID: NCT02345408). So far no clinical trials are underway for CCR1 antagonists in a cancer setting, however, a preclinical study indicates that CCR1 antagonist (BL5923) can suppress metastatic tumor growth of colon cancer cells in the liver [32]. A recent report also shows that another CCR1 antagonist (CCX721) reduces tumor burden and osteolysis in a mouse model of multiple myeloma bone disease [33]. Collectively, these data imply that therapeutic inhibition of CCR1 and CCR2 could be a novel strategy to prevent metastatic tumor growth. *Ccr1Ccr1*^−/−−/−−/−−/−^ mice are healthy without any overt hematopoietic abnormalities unless challenged with specific pathogens [34], suggesting that targeting CCR1 will not cause serious side effects. In contrast *Ccr2Ccr2*^−/−−/−−/−−/−^ mice show a reduced number of circulating monocytes [35] perhaps indicating that CCR2 is a less attractive target than CCR1. However a CCR2 antagonist (CCX140-B) shows therapeutic effect on type II diabetes in a phase II trial without affecting the blood monocyte count [36], suggesting that targeting CCR2 could remain as an important therapeutic strategy for metastatic cancer.

Despite this optimism a treatment with single chemokine antagonist will almost certainly not be enough to suppress metastatic tumor growth since neither *Ccr1Ccr1*Ccr1 and *Ccr2Ccr2*Ccr2 deficiency can achieve complete elimination of metastatic tumors in the mouse model (maximum reduction rate was 60%) and model dependent [13]. There might be two major reasons accounting for this insufficiency, i.e., a redundancy of the target receptors and a lack of direct effects on cancer cells. As described above, it is possible that multiple chemokine receptors such as CCR1, CCR2, CCR5, CCR6, CXCR2, CXCR4, and CX3CR1 can be involved in the MAM accumulation. Interestingly, several studies have suggested that in vitro monocyte migration induced by CCR1 or CCR2 activation is synergistically enhanced by activation of CXCR4 [37,38]. It is therefore possible that the above-mentioned receptors, in particular CXCR4, cooperate with CCR1 and/or CCR2 to promote MAM accumulation and thereby metastatic tumor growth. Another aspect to be considered is that tumor metastasis is supported by MAMs as well as other immune cell types such as myeloid-derived suppressor cells (MDSCs) and regulatory T (T_reg_) cells that are recruited via different chemokine signals [6]. For example, T_reg_ cells migrate towards primary ovarian tumors via CCL22-CCR4 signal [39] and MDSCs accumulate in ovarian caner and sarcoma by CXCL12 (a CXCR4 ligand) and CXCL8 (a ligand for CXCR1/2) [40,41]. In the mammary tumors developed in mice, CXCL5 (a CXCR2 ligand) and CCL5 (a ligand for CCR1/3/5) can promote infiltration of MDSCs and T_reg_ cells respectively [42,43], although their roles at metastatic sites remain to be identified. Considering such chemokine receptor redundancies, it may be necessary to block multiple chemokine signals to achieve full therapeutic effects. This strategy seems to be practical since it is possible to design a drug that can target more than one chemokine receptor. So far, dual-antagonists for CCR1/CCR3, CCR2/CCR5, and CCR2/CXCR2 have been developed and tested in animal models of acute and chronic inflammation [31]. However, clinical application for metastatic breast cancer requires more investigations of these synergies to identify combination of chemokine signals that promotes metastatic tumor growth and to elucidate therapeutic effects of the concomitant blockade of these signals.

Although breast cancer cells also express chemokine receptors including CCR5, CCR7 and CXCR4 that enhance tumor cell invasiveness and metastasis [44], it is unlikely that a single treatment with chemokine antagonist can directly induce tumor cell death. It will thus be essential to combine chemokine antagonists that target stromal cells with therapeutic modalities such as chemotherapy or immunotherapy that directly kill the cancer cells. Several studies report that blockade of myeloid cell recruitment by chemokine antagonists synergistically enhances the therapeutic efficacy of cytotoxic drugs. For example, genetic loss of host CCR2 expression suppresses monocyte accumulation and enhances the effect of doxorubicin or cisplatin treatment on the relapse of mammary tumors in the PyMT mice [19]. A CCR2 antagonist (PF-04136309) also suppresses macrophage accumulation in the primary tumor developed by orthotopically injected pancreatic cancer cells, which enhances the effects of gemcitabine on the tumor growth [45]. Furthermore, a CXCR4 antagonist (AMD3100) prevents macrophage accumulation and delays tumor relapse after cyclophosphamide treatment in subcutaneously transplanted lung cancer and in orthotopic mammary cancers [46], and a CXCR2 (SB-265610) antagonist enhances therapeutic effect of a doxorubicin/cyclophosphamide combination treatment in a orthotopic breast cancer xenograft model probably through inhibition of myeloid cell accumulation [47]. Inhibition of monocyte/macrophage accumulation may also improve immunotherapy efficacy. In a pancreatic cancer mouse model, pharmacological macrophage depletion enhances tumor reduction induced by antibodies against T cell inhibitory receptors cytotoxic T-lymphocyte- associated antigen 4 (CTLA4) and programmed cell death protein 1 (PD1) that augment tumoricidal CD8 ^+^ T cell responses [48]. It is also reported that genetic depletion of CCR2^+^ monocytes (CD11b^+^Ly6C^+^) enhances accumulation of adoptively transferred CD8^+^ T cells in the primary tumor and thereby augments therapeutic effect of the adoptive T-cell therapy on the tumor growth in a melanoma model [49]. Based on these findings, it appears feasible to suppress metastatic tumor growth by combination therapy utilizing chemokine antagonists and cytotoxic drugs or immunotherapeutic agents. Nevertheless, in order to bring these approaches into practical application, further studies are required to elucidate the effects of macrophage-mediated chemokine signals on the formation of chemoresistant or immunosuppressive microenvironment at the metastatic sites.

## 4 Conclusion

**Fig. 2:**
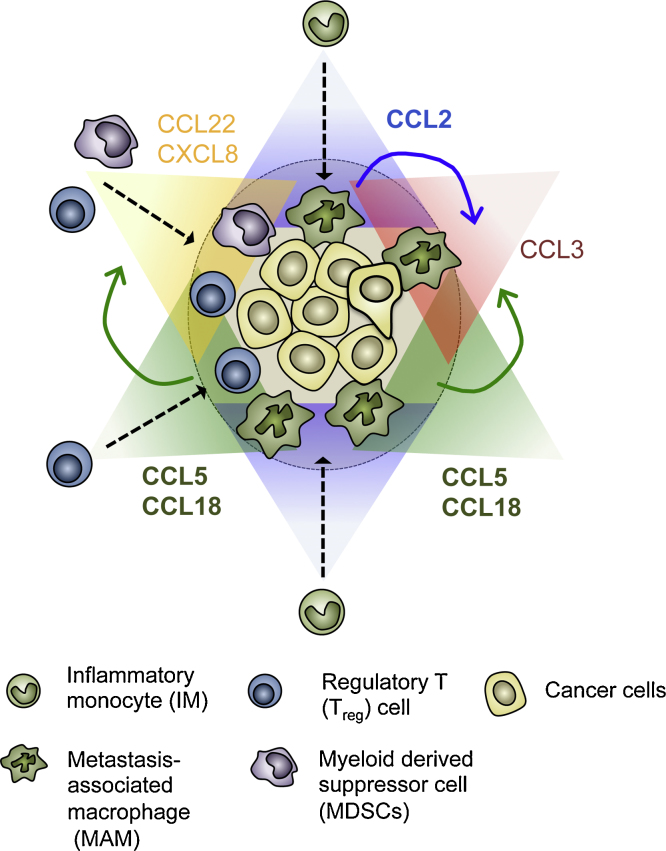
Model for a chemokine network in the metastatic tumor microenvironment. Chemokine gradients of CCL2, CCL5, and CCL18 formed by cancer cells or stromal cells not only recruit IMs and T_reg_ cells toward the tumor microenvironment but also establish another gradient of chemokine ligands (shown in different colors) such as CCL3, CCL22, and CXCL8 that augments the accumulation of MAMs, T_reg_ cells, and MDSCs. These overlapping gradients result in an evolving microenvironment that enhances tumor cell survival, growth and prevents immune attack. Consequently disruption of these gradients suggests a possible therapeutic strategy for metastatic disease.

Our data suggest that a gradient of CCL2 attracts monocytes/macrophages towards the metastatic tumor microenvironment where they are exposed to high levels of CCL2 and are prompted to secrete another chemokine CCL3. As mentioned above, such a chemokine-induced chemokine secretion is also reported in human monocyte culture systems, i.e., exposure to CCL5 or CCL18 induce secretion of CCL2, CCL3, CCL22, and CXCL8 [29,30] that can regulate accumulation of MAMs, MDSCs and T_reg_ cells. Interestingly, mouse breast cancer cells used in our metastasis model can promote CCL5 expression in cultured macrophages (unpublished data). Furthermore, GM-CSF produced by human breast cancer cell lines induces CCL18 secretion from cultured macrophages [50], suggesting that macrophage-cancer cell interaction establish gradients of these chemokines in the tumor microenvironment. Collectively these data suggest that gradients of chemokines (e.g., CCL2, CCL5, and CCL18) formed in the tumor microenvironment not only recruit monocytes/macrophages but also form de novo chemokine gradients (e.g., `, CCL22, and CXCL8) that reinforce the accumulation of pro-metastatic immune cells such as MAMs, MDSCs and T_reg_ cells (Fig. 2). Since these pro-metastatic chemokine signals are also involved in the pathology of chronic inflammatory diseases, many companies have been trying to develop selective or dual-blocking chemokine antagonists for chronic inflammatory diseases. To apply these drugs for metastatic breast cancer, it is necessary to more clearly elucidate the pro-metastatic chemokine network in the sites of metastasis and identify key links and synergistic effects within these gradients that are required for metastatic cells to alter their microenvironment to prosper.

## Conflict of interest

Dr. Pollard has patents pending on targeting CCR1 at metastatic sites.
